# Modeling and equilibrium studies on the recovery of praseodymium (III), dysprosium (III) and yttrium (III) using acidic cation exchange resin

**DOI:** 10.1186/s13065-022-00830-0

**Published:** 2022-05-25

**Authors:** B. A. Masry, E. M. Abu Elgoud, S. E. Rizk

**Affiliations:** Chemistry of Nuclear Fuel Department, Hot Laboratories Centre, Egyptian Atomic Energy Authority, Cairo, Egypt

**Keywords:** Modeling, Dowex 50WX8, Adsorption, Rare earth metals

## Abstract

In this research, the possibility of using hydrogenated Dowex 50WX8 resin for the recovery and separation of Pr(III), Dy(III) and Y(III) from aqueous nitrate solutions were carried out. Dowex 50WX8 adsorbent was characterized before and after sorption of metal ions using Fourier-transform infrared spectroscopy (FT-IR), Scanning Electron Microscope (SEM) and Energy Dispersive X-Ray Analysis (EDX) techniques. Sorption parameters were studied which included contact time, initial metal ion concentration, nitric acid concentration and adsorbent dose. The equilibrium time has been set at about 15.0 min. The experimental results showed that the sorption efficiency of metal ions under the investigated conditions decreased with increasing nitric acid concentration from 0.50 to 3.0 M. The maximum sorption capacity was found to be 30.0, 50.0 and 60.0 mg/g for Pr(III), DY(III) and Y(III), respectively. The desorption of Pr(III) from the loaded resin was achieved with 1.0 M citric acid at pH = 3 and found to be 58.0%. On the other hand, the maximum desorption of Dy(III) and Y(III) were achieved with 1.0 M nitric acid and 1.0 M ammonium carbonate, respectively. The sorption isotherm results indicated that Pr(III) and Y(II) fitted with nonlinear Langmuir isotherm model with regression factors 0.995 and 0.978, respectively; while, Dy(III) fitted with nonlinear Toth isotherm model with R^2^ = 0.966. A Flow sheet which summarizes the sorption and desorption processes of Pr(III), DY(III) and Y(III) using Dowex 50WX8 from nitric acid solution under the optimum conditions is also given.

## Highlights

*The maximum adsorption capacities of Dy(III), Pr(III) and Y(III) were 30, 50 and 60 mg/g respectively using 500 mg/L of Dowex 50WX8 resin.

*Maximum stripping (58%) of Pr(III) was achieved using 1 M Citric acid at pH 3.

*The maximum separation ratios obtained were 56, 35 and 4.6 for Y(III)/Pr(III), Y(III)/Dy(III) and Dy(III)/Pr(III), respectively.

## Introduction

Yttrium (Y), Dysprosium (Dy) and Praseodymium (Pr) known as segment of the rare earth elements (REEs) are spirited components in fluorescent lamps, glass polishing and ceramics, computer monitors, lighting, radar, televisions, and X-ray intensifying films. Yttrium is extensively used in the manufacturing of several high-tech-devices such as microwave communication for satellite industries, color televisions, computer monitors and temperature sensors [1, 2]. Praseodymium is used with neodymium in combination for goggles to shield glassmakers against sodium glare, permanent magnets and cryogenic refrigerant [3]. Dysprosium alloy with neodymium is used for permanent magnets, catalysts, speakers, compact discs and hard discs and medium source rare-earth lamps within the film industry. [4] Ion exchange separation of rare earth elements was used by Spedding and Powell to separate REEs from fission products obtained from nuclear reactors [5–7]. Sorption processes for the separation of rare earths have been reviewed in several articles [8–11]. Strongly acidic cation exchangers were the first artificial functional polymers used for the separation of REE ions, and they are resumed predominating in the fields of chemistry and chemical technology under consideration [11]. Styrene and divinylbenzene copolymers bearing SO_3_H group are utilized. Now, modifications of these exchangers are manufactured under the trade names Amberlite, Dowex, Lewatit, Purolit, which differ by the degree of cross linking sorption capacity, grain size, pore diameter, and other parameter [12]. Dowex 50 W-X8 is one of the ion-exchange resins that have been used for separation of REEs from other ions as well as separation of individual REEs from a mixture of REEs. Al-Thyabat and Zhang**,** studied the recovery of REEs resulting from phosphoric acid with Dowex 50WX4 and Dowex 50WX8 resins. Their results indicated that the REE-extraction efficiency of Dowex 50WX8 was almost twice that of Dowex 50WX4 resin. This can be explained by the higher exchange capacity, producing more sulfonic groups, of Dowex 50WX8 even though its lower surface area and larger bead size [13]. Felipe et al., studied the recovery of rare earth elements from acid mine drainage by ion exchange [14] and reported that the highest loading capacities were 0.212 mmol g^−1^ for La and 0.169 mmol g^−1^ for Ce (Dowex 50WX8) and 0.210 mmol g^−1^ for La and 0.173 mmol g^−1^ for Ce (Lewatit MDS 200 H). Recovery of rare earth elements from uranium concentrate by using cation exchange resin (Dowex 50WX8) was studied and the authors reported that the maximum REE sorption capacity was found to 82.74 mg/g which represents about 93.23% of the original capacity of the studied resin [15]. Sorption of rare earth elements from nitric acid solution with macroporous silica-based bis(2-ethylhexyl)phosphoric acid impregnated polymeric adsorbent has been studied by Shu et al. [16] Their results indicated that the adsorption capacity of Gd (III) was found to be 0.315 mmol g^−1^ by bis(2-ethylhexyl)phosphoric acid/SiO_2_-P in 0.1 M HNO_3_. Adsorption and separation of terbium(III) and gadolinium(III) from aqueous nitrate medium has been investigated using TVEX-PHOR resin by Madbouly et al. [17]. Their work showed that the maximum sorption capacity of this material was 15.49 mg/g and 24.93 mg/g for Gd(III) and Tb(III) from 0.1 M NaNO_3_ solution, respectively at pH = 5.2 and V/m = 0.1. El-Dessouky et al. studied the sorption of praseodymium (III), holmium (III) and cobalt (II) from nitrate medium using TVEX–PHOR resin [18] and reported that 85% sorption was achieved for holmium (III), 75% for praseodymium (III), and 12% for cobalt (II) which enables the possibility of separation of cobalt (II) from the investigated lanthanide elements. The extraction and separation of some rare earths from nitric acid solutions by Cyanex 272 impregnated XAD-7 resin has been examined by İnan et al. [19] The obtained results indicated that REEs have a tendency to behave as two different groups that can be separated into two fractions as La, Pr, Nd and Sm, Eu, Gd. Dowex 50wx8 was used previously for the reversible ion exchange of cerium (III) sulfate and Cerium (III) nitrate where the experimental results indicate that the continuous liquid flow reactor studies show a capacity of 0.72 mmol/g sorbent for the Ce nitrate and 0.96 mmol/g sorbent for the Ce sulfate [20].

The main objective of the present work is directed to study the sorption and separation of Praseodymium, Dysprosium and Yttrium from nitric acid solution using strongly acidic cationic exchange resin (Dowex 50WX8) using batch technique. The effects different parameters on the sorption and separation processes will be investigated such as contact time, nitric acid concentration, as well as v/m ratio and temperature. Desorption investigations will be also carried out and evaluated. Separation feasibility between the investigated REEs are also discussed based on the difference between their sorption and desorption behavior.

## Experimental

### Materials and chemicals

**Fig.1:**
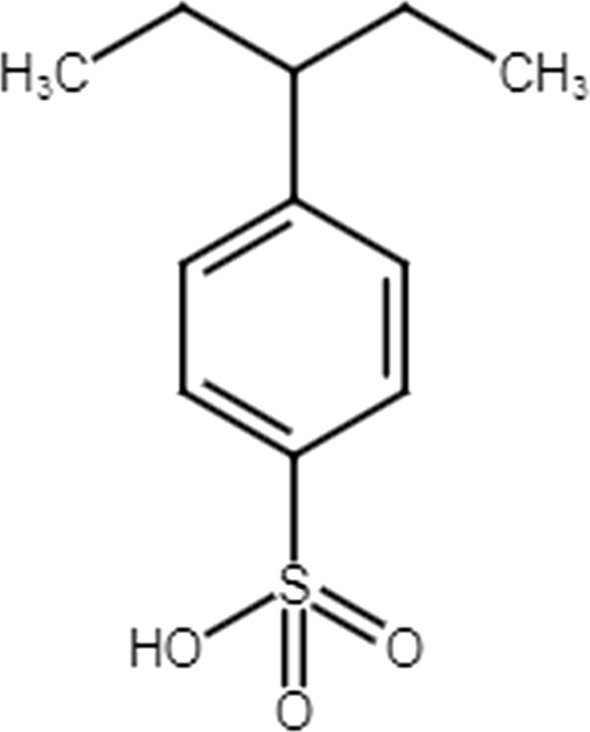
Chemical structure of hydrogenated Dowex 50WX8 resin

The chemicals used in this work were of analytical reagent grade (AR) and most of them were used without further purification. Stock solutions of Pr(III), DY(III) and Y(III) (1000 mg/L) were prepared by dissolving a known amount of the metal oxide in minimum concentrated nitric acid and evaporated to near dryness and then made up to the mark in a measuring flask with double distilled water. The desired required concentrations of test solutions were prepared by favorable dilution with a known concentrated nitric acid of the stock solutions. Dowex 50WX8 which is a strong acid cation resin containing 8% divinylbenzene (DVB) [20], Fig. 1 was purchased from sigma Aldrich. The chemical and physical specifications of Dowex 50X8 are given in table given in Table 1.

**Table 1:** Specification data sheet of Dowex 50WX8

Product specification	
Appearance (Color)	Faint Yellow to Brown
Appearance (Form)	Beads
Chemical form	Strongly acidic hydrogen form containing 8% divinylbenzene (DVB)
Mesh size	100–200 mesh
Particle size	Distribution > 90% within 300 to 1180 UM
Wet capacity	> 1.7 _MEQ/ML
Moisture content	50–60%

### Sorption experiments

The sorption experiments were carried out under the following conditions, v/m = 0.05 L/g, Pr(III), DY(III) and Y(III) concentrations = 100.0 mg/L in 0.50 M nitric acid solution. In each adsorption experiment, 5.0 ml of the investigated metal ions solution was added to 0.1 g of Dowex 50WX8 resin (100–200 mesh) in stoppered glass bottles which were then shaken at (25 ± 1 °C) in a water thermostatic shaker. The concentrations of Pr(III), Dy(III) and Y(III) ions were measured using UV-visible spectrophotometer (a Shimadzu UV-160, Japan) with Arsenazo (III) method [21, 22], and the adsorption capacity (q_e_) at equilibrium was given by equation:

1 $$ q_{e} = \,(C_{i} - C_{e} ) \times \left( {\tfrac{V}{w}} \right)\,\,\,\,\,\,\,\,\,\,\,\,\,\,\,\,\,\,\,\,\,\,\,\,\,\,\,\,\,\,\,\, $$

The sorption efficiency (S %) at equilibrium was calculated from the equation:

2 $$ S\% \, = \,\tfrac{{C_{i} - C_{e} }}{{C_{{_{e} }} }}\,\,\, \times 100\,\,\,\,\,\,\,\,\,\,\,\,\,\,\,\,\,\,\,\,\,\,\,\,\,\,\,\,\,\,\,\,\,\,\,\,\,\,\,\,\, $$

where ${C}_{i}$ and ${C}_{e}$ are the initial and equilibrium metal ions concentrations (mg/L) of metal ions, respectively; v is the volume of the used aqueous solution in liter (L) and w is the weight of the adsorbent (g).

### Characterization techniques

Scanning electron microscopy coupled with energy dispersive X-ray spectroscopy (SEM/EDX) was used to examine the morphology and determine the elemental composition of the metal ions bonding to Dowex 50WX8 resin under the used experimental conditions. The functional groups included in the used adsorbent were investigated using Fourier transform infrared (FT-IR) spectroscopy (Bruker) in the scanning range of 4000–400 cm^−1^ and pH was measured using Hannah pH meter.

### Desorption investigations

Various reagents such as mineral acids, sodium carbonate, ammonium carbonate and citric acid (at different pH) were used for the desorption investigations of the metal ions under study. In this context, 0.1 g of Dowex 50WX8 loaded with about 100.0 mg/L of each individual Dy(III) or Pr(III) or Y(III) was shaken with 5.0 mL of the stripping solution for 20.0 min under the same sorption experimental conditions.

## Results and discussion

### Characterization of Dowex 50WX8

**Fig. 2:**
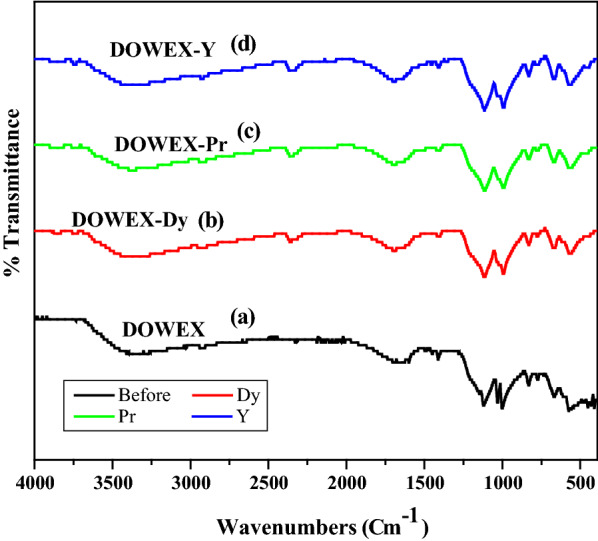
FT-IR spectra of, **a** Dowex 50WX8, **b** Dowex -Dy, **c** Dowex -Pr, **d** Dowex -Y for sorption of rare earth metal ions from nitrate medium

Figure 2a–d shows the FT-IR spectra of Dowex 50WX8 before and after loading of rare earth metal ions (Pr(III), DY(III) and Y(III)), The spectrum of (Pure Dowex 50WX8) showed adsorption peaks in the ranges of 3330–3450 cm^−1^ and 1632–1645 cm^−1^, which may be attributed to stretching vibrations of the O–H functional group in the structure of the adsorbents [20]. Moreover, bands at 1658–1648 cm^−1^ correspond to the alkene group (C=C) in the Dowex skeleton and bands at1350–1340 cm^−1^ are assigned to the stretching S=O of sulfonic acid. Dowex 50WX8 gives characteristic IR bands for the SO_3_ vibration (1169 cm^−1^), (S–C) vibration (1121 cm^−1^), and (S–O) vibrations (1093 and 1037 cm^−1^) [23]. Previous studies have shown that hydration of the sulfonate site with H_2_O leads to the formation of hydronium ion (H_3_O^+^‏) species which is the interaction moiety with metal ions.

The FT-IR spectrum of Dowex after interaction with REE metal ions, indicate that adsorbing species (Dowex/Pr/Dy/Y) were formed at the counter ion of the Dowex 50WX8 (H^+^) which protonates with H_2_O to form the Dowex 50WX8(H_3_O^+^) species [20].

**Fig. 3:**
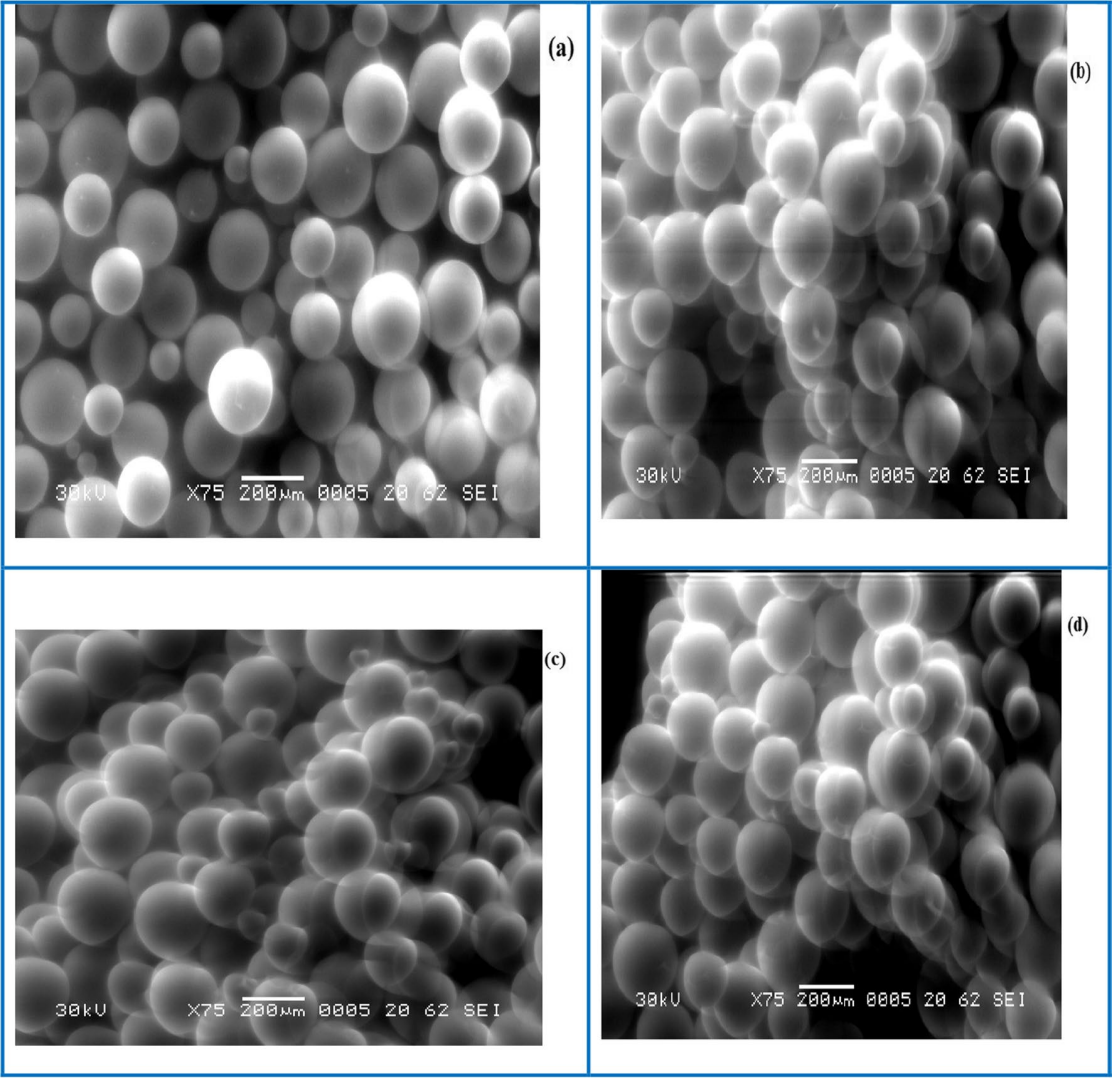
SEM images of, **a** Dowex 50WX8 before sorption process, **b** after sorption of Pr(III), **c** after sorption of Dy(III), **d** after sorption of Y(III) from nitrate medium

The morphology investigations and particle surface variations of the sorbent were given by EDX. Map and SEM analyses were performed both before and after the adsorption of metal ions, Fig. 3a–d. The obtained results show the presence of a variety of pores with a wide range of pore size on the surface of the Dowex 50WX8 resin; the pore space could be attributed to the adsorption process.

**Fig. 4:**
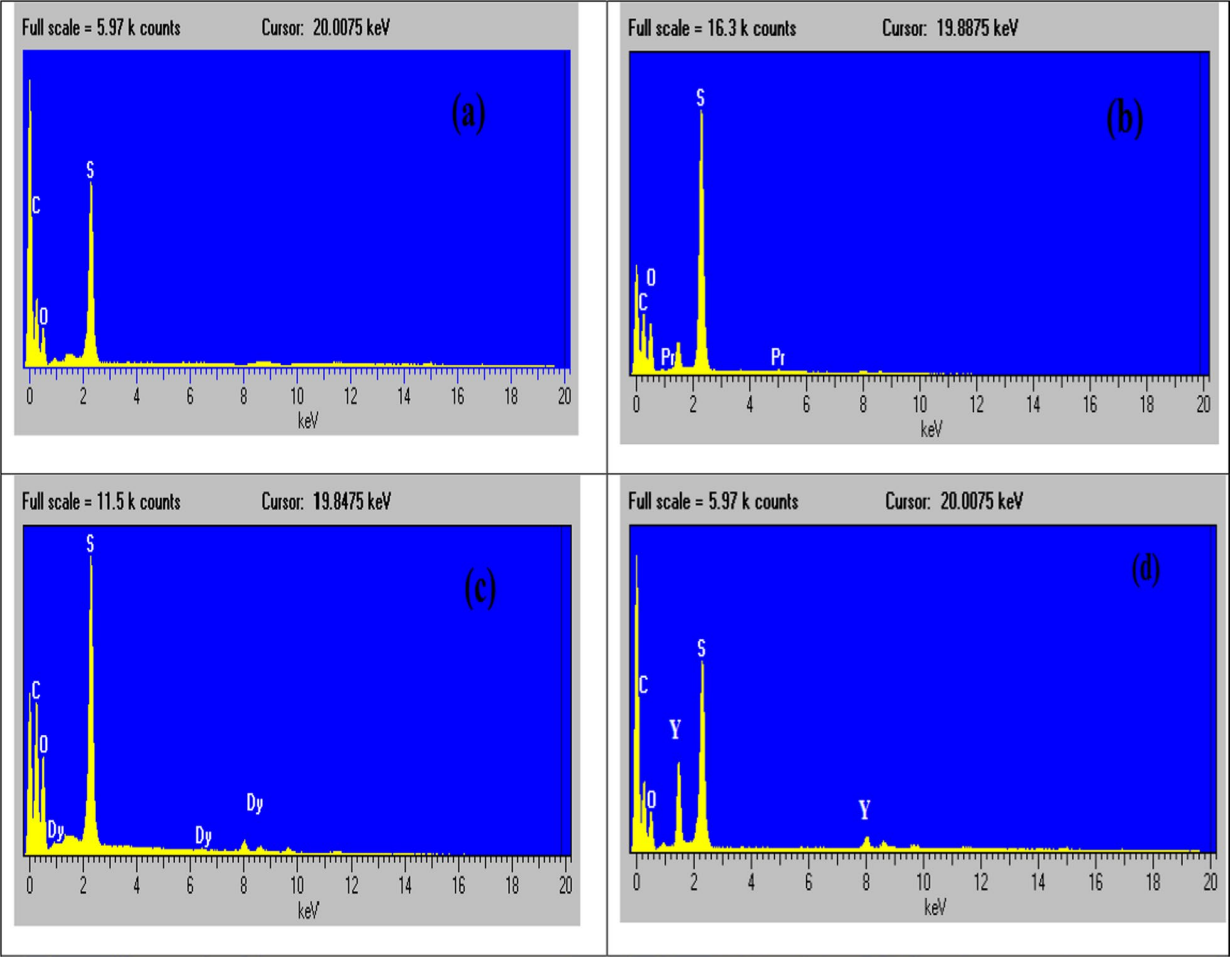
EDX-analysis of **a** Dowex 50WX8 before sorption process, **b** after sorption of Pr(III), **c** after sorption of Dy(III), **d** after sorption of Y(III) from nitrate medium

The results of the EDX and Map analyses indicate the presence of different elements on the surface of the Dowex 50WX8, including Praseodymium, Dysprosium and yttrium, with the elements distributed uniformly across the resin surface Fig. 4(a–d). Consequently, the results of the EDX and Map analyses affirm the successful bonding of the metal ions on the surface of Dowex 50WX8.

### Sorption batch experiments

#### Effect of shaking time

**Fig. 5:**
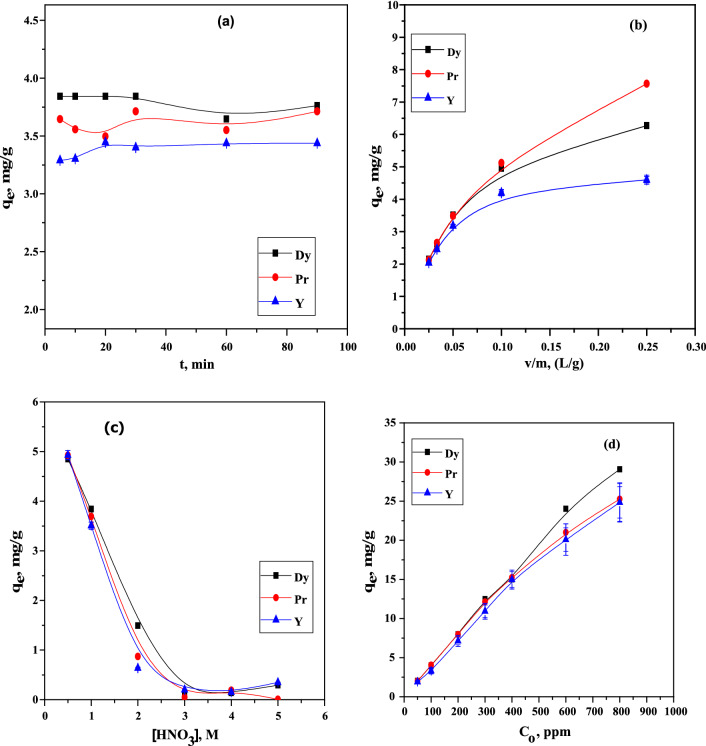
**a** Effect of contact time on the adsorption efficiency of Pr(III), Dy(III) and Y(III) using Dowex 50WX8 from nitrate medium (temperature: 25 °C, initial ion concentration: 100 mg/L, v/m = 0.05 L/g, **b** Effect of the sorbent dosage on the adsorption efficiency (at optimal pH value, contact time = 15.0 min, initial metal ion concentration 100 mg/L, temperature: 25 °C, **c** Effect of nitric acid concentration, **d** Effect of metal ion concentrations on the sorption of Dy, Pr and Y using Dowex 50WX8

The impact of contact time on the sorption efficiency of Pr(III), Dy(III) and Y(III) ions using Dowex 50WX8 sorbent from nitrate medium was carried out in the range 1.0–90.0 min. The results indicated that the rare earth ions sorption process took 1–15 min to occur and was based on the availability of vacant active sites The rate of adsorption on the surface of the adsorbents was significantly decreased at contact times beyond 15.0 min, Fig. 5a possibly because of the saturation of the available active sites on the sorbent surface; accordingly the equilibrium contact time for the sorption of the investigated metal ions using Dowex adsorbent was fixed at 15.0 min.

#### Adsorbent dosage

The sorbent dosage is another key factor imposing large contribution to the sorption process, as it can determine the adsorption efficiency of the sorbent for a given initial concentration of the investigated metal ions [24]. In this respect, the impact of the sorbent dosage (v/m) on the adsorption efficiency of Pr(III), Dy(III) and Y(III) ions was investigated in the range of 0.02–0.25 L/g, Fig. 5b. The obtained results show that the adsorption efficiency yield takes the order DY(III) > Pr(III) > Y(III) enhanced abruptly by increasing v/m from 0.1 L/g up to 0.25 L/g. Based on the obtained results, the optimal adsorbent dosage on Dowex was fixed at 0.05 L/g in all experiments carried out in this work.

#### Effect of nitric acid concentration

The adsorption of Pr(III), Dy(III) and Y(III) from different nitric acid concentrations is given in Fig. 5c and the experimental results revealed that the adsorption efficiency of Dowex ion exchanger decreased rapidly upon increasing nitric acid concentration from 0.50 to 3.0 M. This may be attributed to the compensation of H^+^ with higher increasing of acid concentration which leads to a decrease in the exchange rate between hydrogen ion and metal ions; with further increase in the acid molarity higher than 3.0 M the adsorption capacity became stable, Fig. 5c. However, the experiments were performed at 0.5 M HNO_3_.

#### Effect of metal ions concentration

The effect of the initial concentration of Pr(III), Dy(III) and Y(III) on the adsorption capacity (q_e_) was studied in the range of 50–500 mg L^−1^ through their sorption by Dowex from nitrate medium. The experiments were carried out by shaking 5.0 mL of the investigated metal ions solution individually with 0.05 g of the adsorbent for 15.0 min at 25 °C. The obtained data are represented in Fig. 5d, The adsorption efficiency increased as the concentration of REEs^+3^ increased and the highest adsorption capacities of 29.0 mg/g, 25.0 mg/g and 24.0 mg/g were achieved at 500 mg/L for Dy, Pr and Y, respectively. This saturation can be ascribed to the interactions between the adsorbent active sites and these metal ions. [25].

#### Sorption mechanism of REE^+^ with Dowex-H^+^

Based on the experimental results and considering that M (NO_3_)_2_^+^ is the predominant species in 1.0 M nitric acid solution [21, 26], where M represents Pr(III), Dy(III) and Y(III),The ion exchange extraction mechanism of REEs metal ion (M) with Dowex-H was suggested to proceed via different reaction pathways from Eqs. (3) and (4), [20]

3 $$ 3DOWEX - SO_{3} - 3H^{ + } + M\left( {NO_{3} } \right)_{2}^{ + } \rightleftharpoons 2{\text{DOWEX}} - SO_{3} - {\text{M}}NO_{3} {\text{ + HNO3 + }}DOWEX - SO_{3} - H^{ + } \, $$

4 $$ 3DOWEX - SO_{3} - 3H^{ + } + M\left( {NO_{3} } \right)_{2}^{ + } \rightleftharpoons {\text{DOWEX}} - SO_{3} - {\text{M}}NO_{3} { + 2}DOWEX - SO_{3} - 2H^{ + } \, $$

Equation (3) suggests that the extraction mechanism occurs via partial ion exchange reactions during the REE diffusion and interaction at the Dowex active sites SO_3_-H^+^ where the extracted metal ions species according to Eq. (3) were found to be Dowex-SO_3_-Pr(NO_3_), Dowex-SO_3_-Dy(NO_3_) and Dowex-SO_3_-Y(NO_3_) for Pr, Dy and Y respectively.

### Adsorption isotherm of Pr, Dy and Y on the Dowex 50WX8 cation exchanger

Adsorption isotherms were used to describe the distribution of metal ions between the sample solution (liquid phase) and the resin (solid phase) when the ion exchange process reaches equilibrium [27, 28]. The Langmuir isotherm model describes a homogeneous monolayer chemical adsorption process, while the Freundlich isotherm model describes a heterogeneous physical adsorption process [29]. Non-liner models achieved the most flexible curve fitting functionality. In this context, Langmuir, Freundlich, Temkin, D–R isotherm and Toth isotherm were employed for studying the nonlinear adsorption isotherm of Pr, Dy and Y on the cation exchanger resin (Dowex 50WX8).

Nonlinear Langmuir isotherm equation is given as:

5 $$ {\text{q}}_{{\text{e}}} \,{ = }\,{\text{Q}}\frac{{{\text{bC}}_{{\text{e}}} }}{{{\text{1 + bC}}_{{\text{e}}} }} \, $$

where q_e_ is the equilibrium adsorption capacity of ions on the adsorbent (mg g^−1^), C_e_ is the equilibrium ions concentration in solution (mg L^−1^), Q the maximum capacity of the adsorbent. (mg g^−1^), and b the Langmuir adsorption constant (L mg^−1^). Nonlinear Freundlich isotherm equation is given as:

6 $$ q_{e\,} \, = \,K_{f} \,C_{e}^{1/n} \, $$

where K_f_ is the Freundlich constant (mg/g).

Nonlinear Temkin isotherm model which takes into account the interactions of ions of the aqueous solution and the adsorbent and is given as:

7 $$ q_{e} = \frac{RT}{b}\ln (A_{T} C_{e} ) \, $$

where *R* is the universal gas constant (8.314 J/mol K), *T* the absolute temperature, b a constant related to the heat of sorption (J/mol), *A*_*T*_ the equilibrium binding constant (L/g) and b the adsorption constant (J/mol K).

Toth isotherm model is another empirical equation developed to improve Langmuir isotherm fittings and take into consideration both low and high-end boundary of the concentration and is given as, [30, 31]

8 $$ q_{e} = \, q_{m} \exp ( - \beta \varepsilon^{2} ) \, $$

9 $$ \varepsilon = RT\ln \left( {1 + \frac{1}{{C_{e} }}} \right) \, $$

10 $$ E = \frac{1}{{(2\beta )^{0.5} }} \, $$

**Fig. 6:**
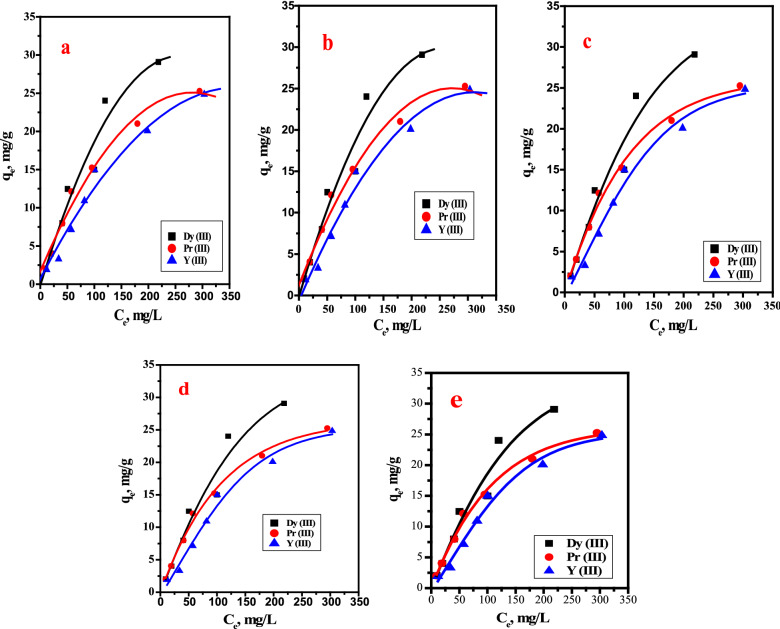
**a** Nonlinear isotherm plot of Langmuir model, **b** Nonlinear isotherm plot of Freundlich model, **c** Nonlinear isotherm plot of D-R, **d** Nonlinear isotherm plot of Temkin model, **d** Nonlinear isotherm plot Toth model for adsorption of Pr(III), DY(III) and Y(III) onto Dowex 50WX8

The relationship between q_e_ and C_e_ for each nonlinear isotherm model is plotted in (Fig. 6 a–e) and the values of the obtained parameters are tabulated in Table 2. The results indicate that Pr and Y fitted with nonlinear Langmuir isotherm model with regression factors 0.995 and 0.978 respectively, while, Dy was fitted with nonlinear Toth isotherm model with R = 0.966.

**Table 2:** Nonlinear Freundlich, Langmuir, Dubinin–Radushkevich, Temkin and Toth isotherm parameters for adsorption of metal ions onto Dowex 50WX8

Isotherm	Parameters	Metal ions		
		DY(III)	Pr(III)	Y(III)
Langmuir	Qo (mg/g)	62.32	36.94	48.76
	b (ml/mg)	0.0041	0.0074	0.0035
	R_L_	0.707	0.575	0.739
	R^2^	0.956	0.995	0.978
	Chi^2	5.36	0.43	1.97
Freundlich	K_f_ (mg/g)	0.632	1.044	0.491
	1/n	0.719	0.569	0.694
	R^2^	0.944	0.971	0.959
	Chi^2	6.765	2.604	3.597
Dubinin–Radushkevich	*q*_*m*_	245.38	116.96	142.26
	*β* mol^2^ /kJ^2^	0.0078	0.0064	0.0086
	*R*^*2*^	0.952	0.484	0.971
	E_DR_	7.996	8.846	7.612
	Chi^2	2.20 E-10	7.11E-11	3.23E-10
Tempkin	$\Delta Q$, kJ/mol	280.66	351.33	328.04
	K_0_, mmol/g	0.0888	0.1017	0.0657
	R^2^	0.8989	0.9738	0.9119
	Chi^2	12.3	2.34	7.77
Toth isotherm	q_T_, mg/g	23.19	25.02	49.11
	K_T_	0.035	0.04	0.017
	N	1.018	1.057	1.048
	R^2^	0.9668	0.987	0.991
	x^2^	1.504	1.19	1.688

## Desorption, reusability and separation between Dy, Pr and Y from nitrate medium using Dowex 50WX8

**Fig. 7:**
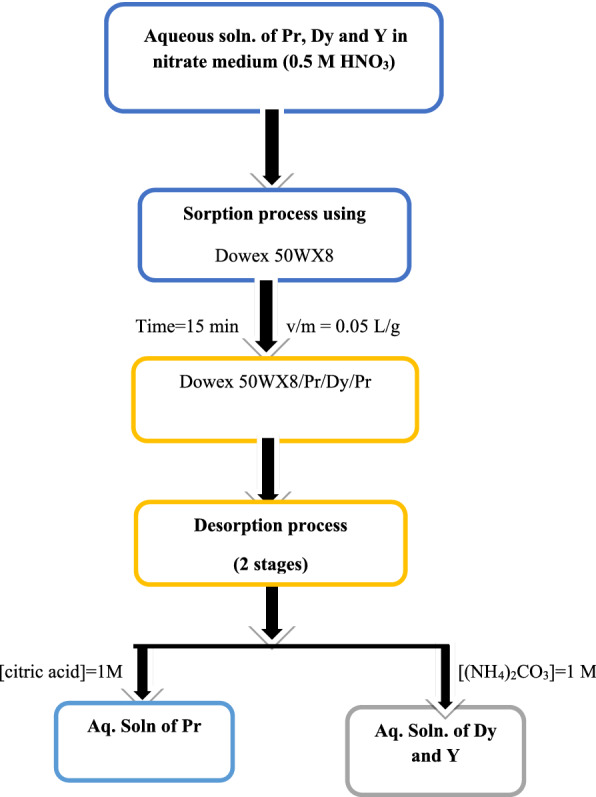
Flow sheet for the sorption and desorption processes of Pr, DY and Y using Dowex 50WX8

The most effective separation obtained between the investigated metal ions was obtained from the stripping process. This process was carried out by contacting the loaded Dowex 50WX8 with different stripping agents at experimental conditions (contact time = 60.0 min, v/m = 0.05 at 25 ± 1 °C). The results illustrated in Table 3 show that the maximum stripping of Pr(III) is 58% and was achieved with 1.0 M citric acid at pH = 3. In the case of Dy(III) and Y(III) the maximum desorption is 55% and 56% and was achieved with [HNO_3_] = 1.0 M and [(NH_4_)_2_CO_3_] = 1.0 M, respectively. A flow sheet which illustrates the sorption and desorption processes of the investigated rare earth using D-50WX8 from 0.5 M HNO_3_ solution at v/m = 0.05 at 25 ± 1 °C is given in Fig. 7.

**Table 3:** Desorption of metal ions (III) with different reagents after their adsorption with the Dowex 50WX8 resin at v/m = 0.05 at 25 ± 1 °C

Stripping agent, M	Dy(III)	Pr(III)	Y(III)	S-ratio		
				Dy/Pr	Y/Pr	Dy/Y
HNO_3_, 1.0 M	24.9	23.7	28	1.05	1.2	1
HNO_3_, 5.0 M	55.4	12	43	4.6	3.6	1.2
HCl, 1.0 M	–	–	7.72	–	–	–
H_2_SO_4_, 1.0 M	32.9	32.6	31	1	–	1.05
Na_2_CO_3_, 1.0 M	21	25.9	20	–	–	1
(NH_4_)_2_CO_3_, 1.0 M	35	–	56	35	56	–
Citric acid (1.0 M)						
pH 1	–	–	–	–		–
pH 3	40.12	58.37	39.25	–		1
pH 5	18.11	35.26	20.46	–		–

The desorption results indicate that Pr(III) can be separated from Dy(III) and Y(III) as follows:

i. Stripping of Dy(III) and Y(III) using [(NH_4_)_2_CO_3_] = 1.0 M from Loaded Dowex 50WX8 after 2 cycles
ii. Dowex 50WX8 containing Pr(III) was then stripped with 1.0 M citric acid at pH = 3 after two stripping cycles

Furthermore, the separation ratio (S-ratio) between the investigated metal ions were calculated by dividing their desorption percentages. The results indicate that the maximum S-ratios are 56.0, 35.0 and 4.6 for Y/Pr, Dy/Pr and were achieved with [(NH_4_)_2_CO_3_] = 1.0 and [HNO_3_] = 5.0 M respectively, Table 3.

Finally, the reported results show that Dowex 50WX8 resin is relatively selective, high efficient and cost effective for Pr(III), Dy(III) and Y(III) adsorption and is also easily regenerated rather than other reported adsorbent/ion exchangers which were used in the adsorption from acidic nitrate medium. The reusability was carried out for 4.0 adsorption stages with sorption capacity of 15.0, 30.0, 35.0 mg/g for Pr, DY and Y, respectively, under the used experimental conditions.

### Comparison study of REEs/Dowex 50WX8 with other reported materials

Comparison of REEs/Dowex 50WX8 system under the used optimum conditions of batch technique with other commercially reported materials [18, 32–51] and given in Table 4, shows the advantages and efficiency of Dowex 50WX8 adsorbent. The results of comparison in the term of maximum capacity (Q_0_) (30, 50, 60 mg/g for Pr, DY and Y), pH = 1, and contact time (15 min) and which were achieved in the current study indicate that Dowex 50WX8 is more efficient and affordable than other reported materials.

**Table 4:** Comparison study of REEs/Dowex 50WX8 with other reported materials

Metal ion	Adsorbent	Q_0_, mg/g	pH	Contact Time	Refs
	Zeolitic imidazolate frameworks nanoparticles	430.4	7.0	7.0 h	[1]
	Oxidized multi-walled carbon nanotubes	78.12	5.0	2.0 h	[2]
	Silica/polyvinyl imidazole/H2PO4-core–shell NPs	150	4.0	0.5 h	[3]
	Hybrid Lewis base ligands functionalized alumina-silica	125.4	4.0	3.0 h	[4]
	polyethylenimine–acrylamide/SiO_2_ hybrid hydrogel	50–100	2-7	6.0 h	[9]
	Microcapsules containing dibenzoylmethane	70.85	6.0	60.0 h	[12]
	D113 resin	292.7	6.0	–	[15]
	Macroporous poly(vinylphosphoramidic acid) resin	101	4-5	–	[14]
	Zr-modified mesoporous silica supported H4[PMo_11_VO_40_]	52.63	5.0	1.0 h	[13]
	Polyacrylic acid grafted silica fume	251.20	1-6	1.0 h	[11]
Pr(III)	Lanthanide-ion imprinted polymers (L-IIPs)	125.3	6.0	1.5 h	[5]
	Polyethylenimine sodium phosphonate resin (PEIPR.Na)	6.23	4.0	250 min	[18]
	Fe_3_O_4_@TiO_2_@P_2_0_4_ nanoparticles	10.2	5.0	–	[19]
	TVEX–PHOR resin	49	3.5	1.0 h	[20]
	magnetic nanoparticles functionalized with a phosphonic group	17.6	4.0	1.0 h	[21]
	silica gel modified with diglycol amic acid	12.72	1.0	–	[22]
	Graphene Oxide Nanosheets	135	6.0	2.0 h	[6]
Y(III)	Graphene oxide nanosheets with cross-linked by high-gluten flour	32.84	7.5	2.0 h	[7]
	Porous three-dimensional graphene oxide-corn zein composites	14.2	–	3.33 h	[8]
	Carbon nanotubes reinforced silica composite	68.8	4.0	24.0 h	[10]
	Functionalized silica in the hybridization process with chitosan	159	4.0	24.0 h	[16]
	Diglycolamic-acid modified chitosan sponges	40.7	0.5–7	12.0 h	[17]

## Conclusions

Dowex 50WX8 was successfully used for the recovery of DY(III), Pr(III) and Y(III) from acidic nitrate medium. The calculated maximum capacity of Dowex 50WX8 is 30, 50, 60 mg/g for Pr, DY and Y respectively at the optimum batch conditions; the maximum stripping of Pr(III) is 58.0% and was achieved with 1.0 M citric acid at pH = 3**.** The results indicate that Pr(III) and Y(III) fitted with nonlinear Langmuir isotherm model with regression factors 0.995 and 0.978 respectively. The regenerated Dowex 50WX8 gave sorption capacities of 15.0, 30.0, 35.0 mg/g for Pr, DY and Y, respectively under the used experimental conditions.

## Declaration

## Data Availability

All data generated or analyzed during this study are included in this published article [and its supplementary information files].

## References

[1] Charalampides G, Vatalis KI, Apostoplos B, Ploutarch-Nikolas B (2015). Rare Earth Elements: Industrial Applications and Economic Dependency of Europe. Procedia Econ Financ.

[2] Humphries M (2013). Rare earth elements: the global supply chain.

[3] Akah A (2017). Application of rare earths in fluid catalytic cracking: a review. J Rare Earths.

[4] Massari S, Ruberti M (2013). Rare earth elements as critical raw materials: Focus on international markets and future strategies. Resour Policy.

[5] Powell JE (1964). The separation of rare earths by ion exchange. Prog Sci Technol Rare Earths.

[6] Spedding FH, Powell EJ (1956). Wheelwright the stability of the rare earth complexes with N-hydroxyethylethylenediaminetriacetic acid. J Am Chem Soc.

[7] JE POWELL 1979Separation chemistry Handbook on the physics and chemistry of rare earths Elsevier 3 81 109

[8] Iftekhar S, Ramasamy DL, Srivastava V, Asif MB, Sillanpää M (2018). Understanding the factors affecting the adsorption of Lanthanum using different adsorbents: a critical review. Chemosphere.

[9] Zubiani EMI, Cristiani C, Dotelli G, Stampino PG (2015). Solid liquid extraction of rare earths from aqueous solutions: a review. Procedia Environ Sci Eng Manag.

[10] Anastopoulos I, Bhatnagar A, Lima EC (2016). Adsorption of rare earth metals: a review of recent literature. J Mol Liq.

[11] Ehrlich GV, Lisichkin GV (2017). Sorption in the chemistry of rare earth elements. Russ J Gen Chem.

[12] Zagorodni AA (2006). Ion exchange materials: properties and applications.

[13] Al-Thyabat S, Zhang P (2015). REE extraction from phosphoric acid, phosphoric acid sludge, and phosphogypsum *Min*. Proc Ext Met.

[14] Felipe ECB, Batista KA, Ladeira ACQ (2021). Recovery of rare earth elements from acid mine drainage by ion exchange. Environ Technol.

[15] Ghazala RA (2015). Recovery of rare earth elements from uranium concentrate by using cation exchange resin. Isot Radiat Res.

[16] Shu Q, Khayambashi A, Wang X, Wei Y (2018). Studies on adsorption of rare earth elements from nitric acid solution with macroporous silica-based bis (2-ethylhexyl) phosphoric acid impregnated polymeric adsorbent. Adsorpt Sci Technol.

[17] Madbouly HA, El-Hefny NE, El-Nadi YA (2021). Adsorption and separation of terbium (III) and gadolinium (III) from aqueous nitrate medium using solid extractant. Sep Sci Technol.

[18] El-Dessouky SI, El-Sofany EA, Daoud JA (2007). Studies on the sorption of praseodymium (III), holmium (III) and cobalt (II) from nitrate medium using TVEX–PHOR resin. J Hazard Mater.

[19] İnan S, Tel H, Sert Ş, Çetinkaya B, Sengül S, Özkan B, Altaş Y (2018). Extraction and separation studies of rare earth elements using cyanex 272 impregnated amberlite XAD-7 resin. Hydrometallurgy.

[20] Miller DD, Siriwardane R, Mcintyre D (2018). Anion structural effects on interaction of rare earth element ions with Dowex 50W X8 cation exchange resin. J Rare Earths.

[21] Aly MI, Masry BA, Gasser MS, Khalifa NA, Daoud JA (2016). Extraction of Ce (IV), Yb (III) and Y (III) and recovery of some rare earth elements from Egyptian monazite using CYANEX 923 in kerosene. Int J Miner Process.

[22] Marczenko Z (1976). Spectrophotometric determination of elements; Ellis Harwood.

[23] RM Silverstein FX Webster DJ Kiemle 2005 Spectrometric Identification of organic compounds Hoboken John Wiley 502 35

[24] Masry BA, Elhady MA, Mousaa IM (2022). Fabrication of a novel polyvinylpyrrolidone/abietic acid hydrogel by gamma irradiation for the recovery of Zn Co Mn and Ni from aqueous acidic solution. Inorg Nano Met Chem,.

[25] El-saied HA, Shahr El-Din AM, Masry BA, Ibrahim AM (2020). A promising superabsorbent nanocomposite based on grafting biopolymer/nanomagnetite for capture of ^134^Cs, ^85^Sr and ^60^Co radionuclides. J Polym Environ.

[26] Masry BA, Aly MI, Khalifa NA, Zikry AAF, Gasser MS, Daoud JA (2015). Liquid–liquid extraction and separation of Pr (III), Nd (III), Sm (III) from nitric acid medium by CYANEX 923 in kerosene. Arab J Nucl Sci Appl.

[27] Edebali S, Pehlivan E (2010). Evaluation of amberlite IRA96 and Dowex 1×8 ion-exchange resins for the removal of Cr (VI) from aqueous solution. Chem Eng J.

[28] Hameed BH, Ahmad AA, Aziz N (2007). Isotherms, kinetics and thermodynamics of acid dye adsorption on activated palm ash. Chem Eng J.

[29] Weber WJ (1972). Physiochemical processes for water quality control.

[30] Toth J (1971). State equation of the solid-gas interface layers. Acta Chim Hung.

[31] Vijayaraghavan K, Padmesh TVN, Palanivelu K, Velan M (2006). Biosorption of nickel (II) ions onto Sargassum wightii: application of two-parameter and three-parameter isotherm models. J Hazard Mater.

[32] Abdel-Magied AF, Abdelhamid HN, Ashour RM, Zou X, Forsberg K (2019). Hierarchical porous zeolitic imidazolate frameworks nanoparticles for efficient adsorption of rare-earth elements. Microporous Mesoporous Mater.

[33] Koochaki-Mohammadpour SMA, Torab-Mostaedi M, Talebizadeh-Rafsanjani A, Naderi-Behdani F (2014). Adsorption isotherm, kinetic, thermodynamic, and desorption studies of lanthanum and dysprosium on oxidized multiwalled carbon nanotubes. J Dispers Sci Technol.

[34] Gargari JE, Kalal HS, Shakeri A, Khanchi A (2017). Synthesis and characterization of Silica/polyvinyl imidazole/H2PO4-core-shell nanoparticles as recyclable adsorbent for efficient scavenging of Sm (III) and Dy (III) from water. J Colloid Interface Sci.

[35] Awual MR, Alharthi NH, Okamoto Y, Karim MR, Halim ME, Hasan MM, Sheikh MC (2017). Ligand field effect for Dysprosium (III) and Lutetium (III) adsorption and EXAFS coordination with novel composite nanomaterials. Chem Eng J.

[36] Yusoff MM, Mostapa NRN, Sarkar MS, Biswas TK, Rahman ML, Arshad SE, Kulkarni AD (2017). Synthesis of ion imprinted polymers for selective recognition and separation of rare earth metals. J Rare Earths.

[37] Ashour RM, Abdel-Magied AF, Abdel-Khalek AA, Helaly OS, Ali MM (2016). Preparation and characterization of magnetic iron oxide nanoparticles functionalized by l-cysteine: Adsorption and desorption behavior for rare earth metal ions. J Environ Chem Eng.

[38] Xu X, Zou J, Teng J, Liu Q, Jiang XY, Jiao FP, Chen XQ (2018). Novel high-gluten flour physically cross-linked graphene oxide composites: Hydrothermal fabrication and adsorption properties for rare earth ions. Ecotoxicol Environ Saf.

[39] Xu X, Jiang XY, Jiao FP, Chen XQ, Yu JG (2018). Tunable assembly of porous three-dimensional graphene oxide-corn zein composites with strong mechanical properties for adsorption of rare earth elements. J Taiwan Inst Chem Eng.

[40] Wang Q, Wilfong WC, Kail BW, Yu Y, Gray ML (2017). Novel polyethylenimine–acrylamide/SiO2 hybrid hydrogel sorbent for rare-earth-element recycling from aqueous sources. ACS Sustain Chem Eng.

[41] Ramasamy DL, Puhakka V, Doshi B, Iftekhar S, Sillanpää M (2019). Fabrication of carbon nanotubes reinforced silica composites with improved rare earth elements adsorption performance. Chem Eng J.

[42] Liang T, Yan C, Li X, Zhou S, Wang H (2018). Withdrawn: Polyacrylic acid grafted silica fume as an excellent adsorbent for dysprosium (III) removal from industrial wastewater. Water Sci Technol.

[43] Kondo K, Umetsu M, Matsumoto M (2015). Adsorption characteristics of gadolinium and dysprosium with microcapsules containing an extractant. J Water Process Eng.

[44] Aghayan H, Mahjoub AR, Khanchi AR (2013). Samarium and dysprosium removal using 11-molybdo-vanadophosphoric acid supported on Zr modified mesoporous silica SBA-15. Chem Eng J.

[45] Guanqyao Z, Zhixing S, Xingyin L, Xijun C (1992). Efficiency of macroporous poly(vinylphosphoramidic acid) resin adsorbing of selected elements and determination of trace dysprosium holmium erbiom and ytterbium in waste water by inductively coupled plasma optical emission spectrometry. Anal Lett.

[46] Wang H, Gao P (2007). Adsorption of d113 resin for dysprosium (III). J Wuhan Univ Technol Mater Sci Ed.

[47] Ramasamy DL, Wojtuś A, Repo E, Kalliola S, Srivastava V, Sillanpää M (2017). Ligand immobilized novel hybrid adsorbents for rare earth elements (REE) removal from waste water: assessing the feasibility of using APTES functionalized silica in the hybridization process with chitosan. Chem Eng J.

[48] Bai R, Yang F, Zhang Y, Zhao Z, Liao Q, Chen P, Cai C (2018). Preparation of elastic diglycolamic-acid modified chitosan sponges and their application to recycling of rare-earth from waste phosphor powder. Carbohyd Polym.

[49] Bendiaf H, Abderrahim O, Villemin D, Didi MA (2017). Studies on the feasibility of using a novel phosphonate resin for the separation of U (VI), La (III) and Pr (III) from aqueous solutions. J Radioanal Nucl Chem.

[50] Yan P, He M, Chen B, Hu B (2017). Fast preconcentration of trace rare earth elements from environmental samples by di (2-ethylhexyl) phosphoric acid grafted magnetic nanoparticles followed by inductively coupled plasma mass spectrometry detection. Spectrochim Acta Part B At Spectrosc.

[51] Gaete J, Molina L, Valenzuela F, Basualto C (2021). Recovery of lanthanum, praseodymium and samarium by adsorption using magnetic nanoparticles functionalized with a phosphonic group. Hydrometallurgy.

[52] Ogata T, Narita H, Tanaka M (2015). Adsorption behavior of rare earth elements on silica gel modified with diglycol amic acid. Hydrometallurgy.

