# Correction: Modeling and equilibrium studies on the recovery of praseodymium (III), dysprosium (III) and yttrium (III) using acidic cation exchange resin

**DOI:** 10.1186/s13065-023-00940-3

**Published:** 2023-04-26

**Authors:** B. A. Masry, E. M. Abu Elgoud, S. E. Riz

**Affiliations:** Chemistry of Nuclear Fuel Department, Hot Laboratories Centre, Egyptian Atomic Energy Authority, Cairo, Egypt

**Correction: BMC Chemistry (2022) 16:37**

10.1186/s13065-022-00830-0

Following publication of the original article [1], the author noticed the errors in Table 4 and in the reference list. These have been corrected with this erratum.

In section, “Comparison study of REEs/Dowex 50WX8 with other reported materials”, the paragraph should read “Comparison of REEs/Dowex 50WX8 system under the used optimum conditions of batch technique with other commercially reported materials [32–53] and given in Table 4 shows the advantages and efficiency of Dowex 50WX8 adsorbent. The results of comparison in the term of maximum capacity (Q_0_) (30, 50, 60 mg/g for Pr, DY and Y), pH = 1, and contact time (15 min) and which were achieved in the current study indicate that Dowex 50WX8 is more efficient and affordable than other reported materials.

**Table 4:** Comparison study of REEs/Dowex 50WX8 with other reported materials

Metal ion	Adsorbent	Q0, mg/g	pH	Contact Time	Ref.
	Dowex 50WX8	50	1	15	Current work
	Zeolitic imidazolate frameworks nanoparticles	430.4	7.0	7.0 h	[32]
	Oxidized multi-walled carbon nanotubes	78.12	5.0	2.0 h	[33]
	Silica/polyvinyl imidazole/H2PO4-core-shell NPs	150.0	4.0	0.5 h	[34]
	Hybrid Lewis base ligands functionalized alumina-silica	125.4	4.0	3.0 h	[35]
Dy(III)	polyethylenimine–acrylamide/SiO2 hybrid hydrogel	50-100	2-7	6.0 h	[36]
	Microcapsules containing dibenzoylmethane	70.85	6.0	60.0 h	[37]
	D113 resin	292.7	6.0	--	[38]
	Macroporous poly(vinylphosphoramidic acid) resin	101	4-5	--	[39]
	Zr-modified mesoporous silica supported H4[PMo11VO40]	52.63	5.0	1.0 h	[40]
	Polyacrylic acid grafted silica fume	251.20	1-6	1.0 h	[41]
	Dowex 50WX8	30	1	15	Current work
	Lanthanide-ion imprinted polymers (L-IIPs)	125.3	6.0	1.5 h	[42]
Pr(III)	Polyethylenimine sodium phosphonate resin (PEIPR.Na)	6.23	4.0	250 min	[43]
	Fe3O4@TiO2@P204 nanoparticles	10.20	5.0	--	[44]
	magnetic nanoparticles functionalized with a phosphonic group	17.6	4.0	1.0 h	[45]
	silica gel modified with diglycol amic acid	12.72	1.0	--	[46]
	Dowex 50WX8	60	1	15	Current work
	Graphene Oxide Nanosheets	135.0	6.0	2.0 h	[47]
Y(III)	Graphene oxide nanosheets with cross-linked by high-gluten flour	32.84	7.5	2.0 h	[48]
	porous three-dimensional graphene oxide-corn zein composites	14.2	--	3.33 h	[49]
	carbon nanotubes reinforced silica composite	68.8	4.0	24.0 h	[50]
	functionalized silica in the hybridization process with chitosan	159	4.0	24.0 h	[51]
	Diglycolamic-acid modified chitosan sponges	40.7	0.5-7	12.0 h	[52]

## Reference List

1. Charalampides G, Vatalis KI, Apostoplos B, Ploutarch-Nikolas B. Rare Earth elements: Industrial Applications and Economic Dependency of Europe. Procedia. Econ Financ. 2015;24:126.

2. Humphries M. 2013 Rare earth elements: the global supply chain.Congressional Research Service. The Library of Congress, Washington.

3. Akah A. Application of rare earths in fluid catalytic cracking: a review. J Rare Earths. 2017;35:941.

4. Massari S, Ruberti M. Rare earth elements as critical raw materials: focus on international markets and future strategies. Resour Policy. 2013;38:36.

5. Powell JE. The separation of rare earths by ion exchange. Progress in the Science and Technology of the Rare Earths. 1964;88:4536.

6. Spedding FH, Powell EJ. 1956 Wheelwright The stability of the rare earth complexes with N-hydroxyethylethylenediaminetriacetic acid,J. Am. Chem. Soc.78 34

7. POWELL J E 1979 Separation chemistry. Handbook on the Physics and chemistry of rare earths 3 81

8. Iftekhar S, Ramasamy DL, Srivastava V, Asif MB, Sillanpää. M 2018 Understanding the factors affecting the adsorption of Lanthanum using different adsorbents: a critical review.Chemosphere204413

9. Zubiani EMI, Cristiani C, Dotelli G, Stampino PG. Solid liquid extraction of rare earths from aqueous solutions: a review. Procedia Environ Sci Eng Manag. 2015;2(3):231.

10. Anastopoulos I, Bhatnagar A, Lima EC. Adsorption of rare earth metals: a review of recent literature. J Mol Liq. 2016;221:954.

11. Ehrlich GV, Lisichkin GV. 2017 Sorption in the chemistry of rare earth elements.Russian Journal of General Chemistry87(6)1220

12. Zagorodni AA. Ion exchange materials: properties and applications. Elsevier; 2006.

13. Al-Thyabat S, Zhang P. 2015 REE extraction from phosphoric acid, phosphoric acid sludge, and phosphogypsum Min. Proc. Ext. Met. 124 143

14. Felipe ECB, Batista KA, Ladeira ACQ. Recovery of rare earth elements from acid mine drainage by ion exchange. Environ Technol. 2021;42(17):2721.

15. Ghazala RA. Recovery of Rare Earth elements from Uranium Concentrate by using Cation Exchange Resin. Isotope and Radiation Research. 2015;47(2):219.

16. Shu Q, Khayambashi A, Wang X, Wei Y. Studies on adsorption of rare earth elements from nitric acid solution with macroporous silica-based bis (2-ethylhexyl) phosphoric acid impregnated polymeric adsorbent. Adsorpt Sci Technol. 2018;36(3–4):1049.

17. Madbouly HA, El-Hefny NE, El-Nadi YA. Adsorption and separation of terbium (III) and gadolinium (III) from aqueous nitrate medium using solid extractant. Sep Sci Technol. 2021;56(4):681.

18. El-Dessouky SI, El-Sofany EA, Daoud JA. Studies on the sorption of praseodymium (III), holmium (III) and cobalt (II) from nitrate medium using TVEX–PHOR resin. J Hazard Mater. 2007;143(1–2):17.

19. İnan S, Tel H, Sert Ş, Çetinkaya B, Sengül S, Özkan B, Altaş. Y 2018 Extraction and separation studies of rare earth elements using cyanex 272 impregnated amberlite XAD-7 resin.Hydrometallurgy, 181156.

20. Miller DD, Siriwardane R, Mcintyre D. 2018 Anion structural effects on interaction of rare earth element ions with Dowex 50 W X8 cation exchange resin.Journal of rare earths, 36(8)879

21. Aly MI, Masry BA, Gasser MS, Khalifa NA, Daoud JA. Extraction of ce (IV), Yb (III) and Y (III) and recovery of some rare earth elements from egyptian monazite using CYANEX 923 in kerosene. Int J Miner Process. 2016;153:71.

22. Marczenko Z. Spectrophotometric determination of elements; Ellis Harwood. Ltd: Poland; 1976.

23. Silverstein RM, Webster FX, Kiemle DJ. 2005 Spectrometric Identification of Organic Compounds. 7th ed. Hoboken: John Wiley & Sons, Inc, 2005:502. 35

24. Masry BA, Elhady MA, Mousaa IM. 2022 Fabrication of a novel polyvinylpyrrolidone/abietic acid hydrogel by gamma irradiation for the recovery of Zn, Co, Mn and Ni from aqueous acidic solution, Inorganic and Nano-Metal Chemistry, DOI:10.1080/24701556.2022.2034860

25. El-saied HA, Shahr El-Din AM, Masry BA, Ibrahim AM. 2020 A promising superabsorbent nanocomposite based on grafting biopolymer/nanomagnetite for capture of ^134^Cs, ^85^Sr and ^60^Co radionuclides.Journal of Polymers and the Environment, 28(6)1749

26. Masry BA, Aly MI, Khalifa NA, Zikry AAF, Gasser MS, Daoud JA. Liquid–liquid extraction and separation of pr (III), nd (III), Sm (III) from nitric acid medium by CYANEX 923 in kerosene. Arab J Nucl Sci Appl. 2015;48(3):1.

27. Edebali S, Pehlivan E. 2010 Evaluation of Amberlite IRA96 and Dowex 1×8 ion-exchange resins for the removal of Cr (VI) from aqueous solution.Chem. Eng. J.161161

28. Hameed BH, Ahmad AA, Aziz N. Isotherms, kinetics and thermodynamics of acid dye adsorption on activated palm ash. Chem Eng J. 2007;133:195.

29. Weber WJ. 1972 Physiochemical processes for water quality control.

30. Toth J. 1971 State equation of the solid-gas interface layers.Acta chim. hung.69 311.

31. Vijayaraghavan K, Padmesh TVN, Palanivelu K, Velan M. Biosorption of nickel (II) ions onto Sargassum wightii: application of two-parameter and three-parameter isotherm models. J Hazard Mater. 2006;133(1–3):304.

32. Abdel-Magied AF, Abdelhamid HN, Ashour RM, Zou X, Forsberg K. Hierarchical porous zeolitic imidazolate frameworks nanoparticles for efficient adsorption of rare-earth elements. Microporous Mesoporous Mater. 2019;278:175–84.

33. Koochaki-Mohammadpour SMA, Torab-Mostaedi M, Talebizadeh-Rafsanjani A, Naderi-Behdani F. Adsorption isotherm, kinetic, thermodynamic, and desorption studies of lanthanum and dysprosium on oxidized multiwalled carbon nanotubes. J Dispers Sci Technol. 2014;35(2):244–54.

34. Gargari JE, Kalal HS, Shakeri A, Khanchi A. Synthesis and characterization of Silica/polyvinyl imidazole/H_2_PO_4_-core-shell nanoparticles as recyclable adsorbent for efficient scavenging of sm (III) and Dy (III) from water. J Colloid Interface Sci. 2017;505:745–55.

35. Awual, M. R., Alharthi, N. H., Okamoto, Y., Karim, M. R., Halim, M. E., Hasan, M.M., … Sheikh, M. C. (2017). Ligand field effect for Dysprosium (III) and Lutetium(III) adsorption and EXAFS coordination with novel composite nanomaterials. Chemical Engineering Journal, 320, 427–435.

36. Wang Q, Wilfong WC, Kail BW, Yu Y, Gray ML. Novel polyethylenimine–acrylamide/SiO_2_ hybrid hydrogel sorbent for rare-earth-element recycling from aqueous sources. ACS Sustain Chem Eng. 2017;5(11):10947–58.

37. Kondo K, Umetsu M, Matsumoto M. Adsorption characteristics of gadolinium and dysprosium with microcapsules containing an extractant. J Water Process Eng. 2015;7:237–43.

38. Wang H, Gao P. Adsorption of d113 resin for dysprosium (III). J Wuhan Univ Technology-Mater Sci Ed. 2007;22:653–6.

39. Guanqyao Z, Zhixing S, Xingyin L, Xijun C. Efficiency of macroporous poly(vinylphosphoramidic acid) resin adsorbing of selected elements and determination of trace dysprosium holmium erbiom and ytterbium in waste water by inductively coupled plasma optical emission spectrometry. Anal Lett. 1992;25:561–72.

40. Aghayan H, Mahjoub AR, Khanchi AR. Samarium and dysprosium removal using 11-molybdo-vanadophosphoric acid supported on zr modified mesoporous silica SBA-15. Chem Eng J. 2013;225:509–19.

41. Liang T, Yan C, Li X, Zhou S, Wang H. Withdrawn: polyacrylic acid grafted silica fume as an excellent adsorbent for dysprosium (III) removal from industrial wastewater. Water Sci Technol. 2018;77(6):1570–80.

42. Yusoff, M. M., Mostapa, N. R. N., Sarkar, M. S., Biswas, T. K., Rahman, M. L., Arshad,S. E., … Kulkarni, A. D. (2017). Synthesis of ion imprinted polymers for selective recognition and separation of rare earth metals. Journal of Rare Earths, 35(2), 177–186.

43. Bendiaf H, Abderrahim O, Villemin D, Didi MA. Studies on the feasibility of using a novel phosphonate resin for the separation of U (VI), La (III) and pr (III) from aqueous solutions. J Radioanal Nucl Chem. 2017;312(3):587–97. 10.1007/s10967-017-5244-8.

44. Yan P, He M, Chen B, Hu B. Fast preconcentration of trace rare earth elements from environmental samples by di (2-ethylhexyl) phosphoric acid grafted magnetic nanoparticles followed by inductively coupled plasma mass spectrometry detection. Spectrochimica Acta Part B: Atomic Spectroscopy. 2017;136:73–80. 10.1016/j.sab.2017.08.011.

45. Gaete J, Molina L, Valenzuela F, Basualto C. Recovery of lanthanum, praseodymium and samarium by adsorption using magnetic nanoparticles functionalized with a phosphonic group. Hydrometallurgy. 2021;105698. 10.1016/j.hydromet.2021.105698.

46. Ogata T, Narita H, Tanaka M. Adsorption behavior of rare earth elements on silica gel modified with diglycol amic acid. Hydrometallurgy. 2015;152:178–82. 10.1016/j.hydromet.2015.01.005.

47. Ashour RM, Abdel-Magied AF, Abdel-Khalek AA, Helaly OS, Ali MM. Preparation and characterization of magnetic iron oxide nanoparticles functionalized by l-cysteine: Adsorption and desorption behavior for rare earth metal ions. J Environ Chem Eng. 2016;4(3):3114–21.

48. Xu X, Zou J, Teng J, Liu Q, Jiang XY, Jiao FP, Chen XQ. Novel high-gluten flour physically cross-linked graphene oxide composites: hydrothermal fabrication and adsorption properties for rare earth ions. Ecotoxicol Environ Saf. 2018;166:1–10. 10.1016/j.ecoenv.2018.09.062.

49. Xu X, Jiang XY, Jiao FP, Chen XQ, Yu JG. Tunable assembly of porous three-dimensional graphene oxide-corn zein composites with strong mechanical properties for adsorption of rare earth elements. J Taiwan Inst Chem Eng. 2018;85:106–14.

50. Ramasamy DL, Puhakka V, Doshi B, Iftekhar S, Sillanpää M. Fabrication of carbon nanotubes reinforced silica composites with improved rare earth elements adsorption performance. Chem Eng J. 2019;365:291–304.

51. Ramasamy DL, Wojtuś A, Repo E, Kalliola S, Srivastava V, Sillanpää M. Ligand immobilized novel hybrid adsorbents for rare earth elements (REE) removal from waste water: assessing the feasibility of using APTES functionalized silica in the hybridization process with chitosan. Chem Eng J. 2017;330:1370–9.

52. Bai, R., Yang, F., Zhang, Y., Zhao, Z., Liao, Q., Chen, P., … Cai, C. (2018). Preparation of elastic diglycolamic-acid modified chitosan sponges and their application to recycling of rare-earth from waste phosphor powder. Carbohydrate polymers, 190, 255–261.
